# 

**Published:** 1874-03

**Authors:** 


### TABLE OF CONTENTS.

ORIGINAL COMMUNICATIONS.

Ingrowing Toe Nail. By J. Q. A. Hudson, M.D. - - - - - 125
Average Weight of Children at Birth. By John H. Booth, M.D. - - 133
Case of Opium Poisoning—Antagonizing Action of Belladonna. By Ely Van DeWarker, M.D. - - - - - - - - - - - - - 136
Hydrocele Cured by one Tapping. By H. B. Lee, M.D. - - - - 139
Belladonna in Aphonia Cases. By T. Curtis Smith, M.D. - - - 141
Abscess of Liver. By John W. Hayne, M.D. - - - - - - - 142
Diphtheria. By J. J. Grace, M.D. A Woman with Three Breasts. By A. R. Kilpatrick, M.D. - - - - - - - - - - - - - 144
Chloral in Hooping-Cough and Asthma; Diabetes Cured by the Exclusive Use of Meat and Lactic Acid. - - - - - - - - - - 145

REMARKS, GLEANINGS AND EXTRACTS.

Deep Injection of Chloroform in Tic Dolereaux; Removal of a Sound Kidney in the Human Subject; Complete Occlusion of the Os Uteri Complicating Labor; New Test for Morphia; Case of Paracentesis Throacis, Recovery; Influence of Alcoholism on Conception; Ergotin in Varix of Pregnancy; Arsenic for the Relief of Asthma; For Gastralgia; Prevention of Mercurial Poisoning. By T. Curtis Smith, M.D. - - - - 146
Correction; The Brain During Sleep; Oxygen in Scarlet Fever; Cerebro Spinal Meningetis; Oxygen in Opium Poisoning; Remedy for Dyspepsia; Epidemic Neuralgia; Barberry as an Antiperiodic; Chloral Hydrate in Pertussis; Tannin as an Antidote to Strychnine. By R. C. Word, M.D. 150
New Views on Diabetes; Chloral Hydrate in Incontinence of Urine; Anæsthetics in Midwifery; Clark's Powders for Intermittent Fever; Strychnia with Atropia in Ophthalmia; Frontal Catarrh; A Good Laxative; Cappillary Bronchitis; The Treatment of Cystitis; New Antidysenteric; Rules for Feeding Babies; Uses of Labarraque's Solution; Oleate of Mercury in Treatment of Ringworm; Iodoform in Venereal Diseases; Treatment of Sloughy Ulcers of the Vulva; Chromic Acid; Cholera Infantum; Pumpkin Seed for Tape-worm; Syrup of Borax; Phosphorus in Neuralgia; Ulcerations in Chronic Diarrhœa; Value of Dry-Cupping; Resina Copaibæ; Remedy for Dysentery or Flux; Bismuth in Skin Diseases; Treatment of Croup; The Best Form for Administering the Phosphates; Quinine by the Rectum; Treatment of Biliary Calculus by Choleate of Soda; Cod-Liver Oil Mixture; Glycerin of Borax in Facial Erysipelas; Remedy for the Rapid Cure of Catarrh; Pruritus Vulvæ; Morphine and Cimicifuga Racemosa in Puerperal Convulsions; Opacities of the Cornea; Domestic Pepsin; Abortive Treatment of Boils; On Aromatic Tincture of Assafœtida; Chloral as an Application in Fetid Ulcers; Glycerite of Lime Used in Burns; Gas in Stomach; Quinine Pill Mass; The Treatment of Burns and Scalds; Epithelioma; Introduction of the Stomach Pump Tube; Lime-water in Stings of Bees and Wasps; Wright's Cure for Headache Following Alcohol Debauch; Treatment of Aged Persons; Lumbricoides; Belladonna in Intestinal Invagination and Hernia; Gout Pills; Depilatory Digitalis an Anaphrodisiac; Iodoform in Diseases of the Throat and Nares; Lotion of Acetic Acid for Baldness; Chronic Catarrh of the Pharynx Treated by Galvano-Caustics; How to Deprive Iodine of its Stain; Psoriasis; Carbolic Acid in Obstinate Leucorrhœa; To Prevent Regors Following Catheterism; Dried Meat for Medical Purposes; Oxide of Silver in Menorrhagia; Impotency; Facial Palsy; Ear-Cough; Spots on the Cornea; Gonorrhea Treated by Creosote; Nervous or Sick Headache; Atropine; Uterine Tonic; Chlorine-Water; A Palliative in Painful Urination; Laxatives; Glycerole for Chapping of the Skin; Enlarged Tonsils; For Ulcers at the Rosevell Hospital; Treatment of Inflamed Piles in the Puerperal Bed; Remedy for Chronic Hoarseness; Nervous Dyspepsias; Sciatica; Antidote to Carbolic Acid; Hæmoptysis; Hydrochlorate of Ammonia in Portal Dropsy. By Local Editors. - - - - - 154

EDITORIAL AND MISCELLANEOUS.

To Advertisers; Premiums, - - - - - - - - 183
Book Notices; Demorest's Monthly; The Tishomingo Medical Association of Mississippi - - - - - - - - - - 185
Lippincott's Magazine; Fluid Extract Gelseminum; Appleton's Journal; More Room. - - - - - - - - - - 186
A Few Words Introductory. - - - - - - - 187
Letter from B. P. Reese - - - - - - - - - 188
Ponce DeLeon Spring; List of Cash Payments to the Record - - 190

